# Gouty Tophi Surrounding the Nonabsorbable Sutures of an Achilles Tendon Repair Surgical Site: A Case Report

**DOI:** 10.1155/2024/8878405

**Published:** 2024-09-20

**Authors:** Arcole Brandon, Robert Rella, Tanner Cox, Jess Mullens

**Affiliations:** ^1^ Frederick P. Whiddon College of Medicine University of South Alabama, Mobile, Alabama, USA; ^2^ Department of Orthopedic Surgery University of South Alabama Health University Hospital, Mobile, Alabama, USA

## Abstract

The formation of gouty tophi surrounding the prior surgical site years after an Achilles tendon repair is an extremely rare presentation for which only three previous cases have been documented in the literature. In this case, we report the presentation of a 53-year-old male who had an Achilles tendon repair two and a half years prior and no clinical history of gout, yet during the necessary revision procedure of his Achilles tendon, he was found to have gouty tophi surrounding the nonabsorbable sutures used during his initial surgical repair. This case presentation and the three prior ones all involve the use of nonabsorbable sutures, and these sutures may potentially serve as a nidus for the formation of gouty tophi years after a surgical procedure, even in patients who do not have a clinical history of gout. It is important for clinicians to be aware of this rare clinical presentation as potential sequelae include infectious processes and the need for revision procedures.

## 1. Introduction

Achilles tendonitis is a common orthopedic problem that involves the Achilles tendon and its insertion site. Achilles tendonitis encompasses the progression of insertional Achilles tendonitis, retrocalcaneal bursitis with formation of a Haglund's deformity, and Achilles tendinopathy. The causes of Achilles tendonitis can be very broad and are usually musculoskeletal in nature. Treatment can be operative or nonoperative, and operative treatment should be reserved for those who fail to improve with nonoperative modalities [1].

Insertional Achilles tendonitis is characterized by pain and tendon thickening at its insertion site on the posterior aspect of the calcaneus and is most common in middle-aged and elderly patients who have a tight heel cord [2]. Recurrent trauma damages the tissues in this area, leading to inflammation and cartilaginous and bony metaplasia. This metaplasia occurs most commonly over the posterosuperior aspect of the calcaneus, creating the enlargement commonly known as a Haglund's deformity [3].

Retrocalcaneal bursitis is a condition associated with Achilles tendonitis. Patients with retrocalcaneal bursitis experience pain and fullness directly over and within 2–3 cm of the Achilles tendon insertion site. The bursa that becomes inflamed is between the posterior aspect of the calcaneus and the anterior aspect of the Achilles tendon. At any stage of Achilles tendonitis, Haglund's deformity is visualized well with plain radiographs. Inflammation of the tendon, its insertion site, and the bursa is best visualized with magnetic resonance imaging (MRI) [4].

Achilles tendinopathy is the breakdown of the collagen fibers comprising the tendon, and it most commonly arises as an overuse injury. It is theorized that tendinopathy is most common 2–6 cm proximal to the tendon's insertion site due to anatomical variants in the tendon's vascularity that create a vascular watershed in that area and make it prone to rupture. The decreased vascularity makes tendon healing less efficacious after microtrauma. This can lead to anaerobic degeneration of the tendon and Achilles tendinosis, which is the thickening of the tendon related to a decrease in blood supply, microtrauma, and aging [1, 2].

Treatment for Achilles tendonitis nearly always begins with the nonoperative management of symptoms. Nonoperative treatment consists of pain management with nonsteroidal anti-inflammatory drugs (NSAIDs), activity and shoe wear modifications, and physical therapy. Eccentric exercises are the most efficient method of strengthening the skeletal muscle and have shown significant improvement in function and symptom severity in patients with Achilles tendonitis. Extracorporeal shock wave therapy has also shown significant benefits. Shoe modifications include heel sleeves, pads, and lifts. In some instances, it may be appropriate to treat patients with an ankle-foot orthotic (AFO) brace for 6–9 months to immobilize the ankle joint. Steroid injections have not shown to be an effective treatment method for Achilles tendonitis and carry a risk of rupture of the Achilles tendon [5, 6].

Operative treatment options include excision of the diseased bursa, debridement of the diseased tendon, calcaneal exostectomy, repair of the Achilles tendon, and in some instances, it can include the transfer of another tendon, most commonly the flexor hallucis longus (FHL), to augment the repair [1]. If it is found that over 50% of the Achilles tendon is affected by the pathologic process, then repair with an accessory tendon is indicated. This can come from the FHL, the flexor digitorum longus (FDL), or an allograft. When resecting diseased Achilles tendons, it is mechanically favorable to perform a superior-inferior incision in line with the fibers of the tendon as opposed to a medial-lateral or oblique incision [1, 4].

Intraoperatively, the Achilles tendon often is elevated away from its calcaneal insertion in order to perform a calcaneal exostectomy and to allow for placement of suture anchors directly into the posterior aspect of the calcaneus. The bony component of the anchor buries in and later integrates into the bone with nonabsorbable sutures that pass through and tighten the Achilles to the calcaneus. As the healing process begins, the Achilles tendon anneals to the calcaneus, and the nonabsorbable sutures stay in place indefinitely [1].

Gout is a chronic disease characterized by intermittent flares of which hyperuricemia is the most important risk factor. The first presentation of gout is usually an acute and severe episode of pain that is normally localized to the first metatarsophalangeal joint that generally lasts 7–14 days, unless treatment is implemented. This is then followed by a pain-free period of resolution. It is a relatively common disease with a prevalence ranging from 0.68% to 3.90%, and it occurs more commonly in men who are either Caucasian or African American. People can develop hyperuricemia from eating excess amounts of red meat or seafood, regularly drinking too much alcohol, or taking medications that cause elevated serum levels of uric acid, such as loop diuretics [7]. Patients get supersaturated levels of uric acid from either the overproduction of uric acid, such as the increased consumption of the types of foods and drinks previously listed, or from the underexcretion of uric acid, such as the decreased kidney function characteristic of chronic kidney disease [8].

The pathophysiology of gout is due to the deposition of monosodium urate crystals in joints that results from a proinflammatory cascade initiated by the interaction of the NLRP3 inflammasome with resident macrophages, and this leads to a response involving multiple inflammatory cytokines that is enhanced by mast cells and neutrophils. Patients who are having a suspected acute gout flare usually present with pain, erythema, and swelling of the suspected joint, and systemic symptoms, such as fever, are also present sometimes. The gold standard for the diagnosis of gout is aspiration of the affected joint. If positive for gout, the joint demonstrates the presence of negatively birefringent needle-shaped crystals under polarizing light microscopy, and the synovial fluid of the affected joint will also usually demonstrate leukocytosis and appear yellow and cloudy. Hyperuricemia alone is suggestive of gout, but it is not sufficient to make the diagnosis. Radiographs are usually normal in the initial presentation of gout or may demonstrate general soft tissue swelling, but advanced gout may demonstrate a sclerotic rim and an overhanging edge characteristic of bone erosion on an x-ray of the affected joint. Dual-energy CT is an imaging modality that can identify deposits of uric acid in patients who have suspected gout [8].

During an acute flare of gout, treatment consists of NSAIDs, colchicine, or corticosteroids. Lifestyle modifications, such as the reduction of obesity and decreased consumption of foods and drinks high in uric acid, are important for preventing the recurrence of gout flares. Urate-lowering drugs are also used to decrease the recurrence of gout flares and include the xanthine oxidase inhibitors allopurinol and febuxostat and probenecid, a medication that increases the renal excretion of uric acid. Preventing the recurrence of gout flares is extremely important in stopping the formation of tophi [8].

Tophi are subcutaneous nodules that consist of a chalk-like substance and are made up of monosodium urate crystals. They can form in locations that are not characteristic of gout flares, such as the ears and finger pads, but they can also form in areas that are more commonly associated with gout flares, such as the first metatarsophalangeal joint [8]. They usually represent long-standing, poorly controlled gout characterized by multiple recurrences of acute flares, but this is not always the case [8, 9]. Tophi can rarely present in patients without a documented history of a gout flare [9]. Furthermore, the formation of tophi around a retained foreign body is also an extremely rare occurrence. Specifically, the presence of gouty tophi surrounding the retained, nonabsorbable sutures years after an Achilles tendon surgical repair has only previously been documented three times [10–12].

This patient's case offers an interesting and very uncommon presentation of gouty tophi that formed around the sutures of an Achilles tendon repair surgical site many years after the prior surgical repair. Interestingly, the patient had no history of any clinical gout attacks. Similar case presentations have only been documented three times previously in a review of the literature, so this case provides for an opportunity to educate clinicians on a rare postoperative complication that has the potential lead to negative patient outcomes if not addressed.

## 2. Case Presentation

The patient is a 53-year-old male with a history of left Achilles tendonitis status post surgical intervention with a Haglund's deformity correction approximately two and a half years prior to presentation and a history of a left fibular fracture status post open reduction internal fixation as well as obstructive sleep apnea and diabetes mellitus type 2. He originally came to the orthopedic surgery office for a chief complaint of bilateral heel pain, worse on the left side, that was partially relieved by meloxicam, physical therapy, and boot immobilization and limited his activities of daily living by causing him pain during his functional activities, such as walking. At that time, the patient initially elected to continue with conservative management and did so for approximately 7 months, but the patient reported that he continually had progressively worsening pain over this period that eventually led to him using a knee scooter to ambulate and caused him to present back to the orthopedic surgery office.

**Figure 1:**
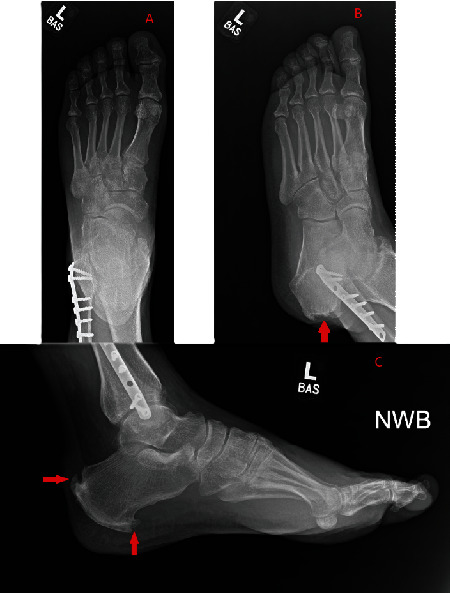
Preoperative radiographs of the left ankle demonstrating anteroposterior (A), oblique (B), and lateral (C) views. Plantar and posterior enthesophytes of the calcaneus are indicated with the arrows.

**Figure 2:**
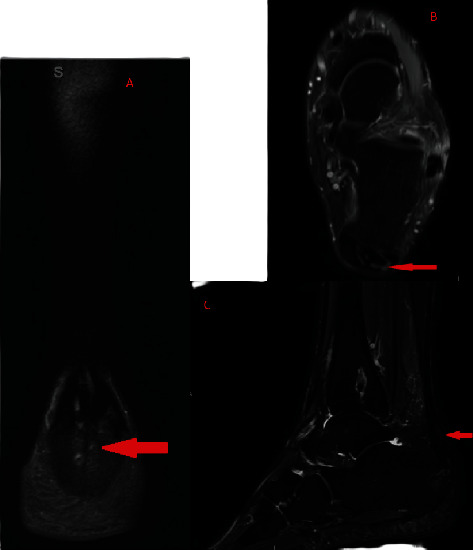
T2 fat suppression MRI with coronal (A), axial (B), and sagittal (C) views of the left ankle with arrows demonstrating thickening and the abnormal signal characteristic of Achilles tendinitis. Notably, there is no radiographic evidence of gouty tophi.

When he presented back to the orthopedic surgery clinic after 7 months of conservative management, his physical exam was significant for tenderness to palpation along the posterior aspect of the heel in addition to evidence of significant swelling and regrowth of bone spurs along the posterior aspect of the Achilles tendon as is demonstrated in Figure 1. There was also evidence of gastrocnemius contracture. The patient was unable to undergo an adequate clinical functional evaluation at this time as the pain was so severe that he was unable to bear any weight on his left leg. While the American Orthopedic Foot & Ankle Society's Ankle-Hindfoot Scale would usually be clinically employed in this patient's case, he was unable to undergo all the necessary exam maneuvers for this clinical tool to be implemented due to severe pain. However, his score would have been very low as he had severe pain almost always present, a severe limitation of his daily activities requiring a scooter to ambulate, and severe difficulty maneuvering on uneven terrain. The patient was told that a revision procedure of his prior left Achilles tendon repair was necessary due to his inability to bear weight on his left leg, the severe restriction the injury put on his lifestyle, and out of a concern that his prior repair had failed, leading to his severe symptoms. The patient consented to the revision procedure and agreed that his lifestyle was significantly restricted due to his injury. Before the revision procedure, the MRI scan that was performed at his initial clinic visit 7 months prior to his current presentation was examined and can be seen in Figure 2. It was significant for the following: “postsurgical changes from prior Achilles tendon repair with tendinopathy and no surrounding edema or associated bursitis.” No imaging findings at this time were suspicious for the presence of any gouty tophi masses.

One week after his return visit to the orthopedic surgery clinic, the patient presented to the hospital for his revision procedure. At the time of the operation, the patient was brought to the operating room and anesthesia was administered without any complications. A tourniquet was used to minimize blood loss for the patient. After the initial incision was made, the surgeons noted that there was a chalky substance that resembled gout buildup surrounding the nonabsorbable sutures of his prior implant, and that substance was removed at this time intraoperatively. The unknown substance was debrided and sent to pathology for further analysis. This intraoperative finding was unanticipated based on the Achilles tendinopathy potentially masking any symptoms of pain in the Achilles tendon these tophi might have caused. Each of the tophi were removed upon discovery after the initial skin incision.

After the unknown substance that was concerning for gouty tophi was debrided and removed, the posterior aspect of the hindfoot was exposed safely before the Achilles tendon was elevated off the calcaneus, the Haglund's deformity was corrected, and the Achilles tendon was debrided to remove unhealthy and scarred tissue. As noted above, the decision to perform an FHL transfer is based on if there is more or less than 50% of viable tendon after debridement and is determined intraoperatively. In this patient's case, it was determined that there was less than 50% of healthy Achilles tendon left, so an FHL tendon transfer was performed. An FHL tendon transfer strengthens the repair of the Achilles tendon and is a preventative health measure to avoid reoperation and decreased function of the graft.

After transfer of the FHL tendon, the patient was not able to reach neutral dorsiflexion, so a gastrocnemius recession was performed to achieve dorsiflexion of around 15°. The operative field was thoroughly irrigated with normal saline to eradicate any debris, including that from the unknown chalky substance that resembled a gout buildup. At the end of the case, the surgeons performed a final check to ensure that the strength of the construct was sufficient to likely avoid further operation in the future. This check was satisfactory, so tranexamic acid was applied to the operative field to assist with hemostasis, and the wound was closed. The patient experienced an uneventful postoperative recovery and discharge.

**Figure 3:**
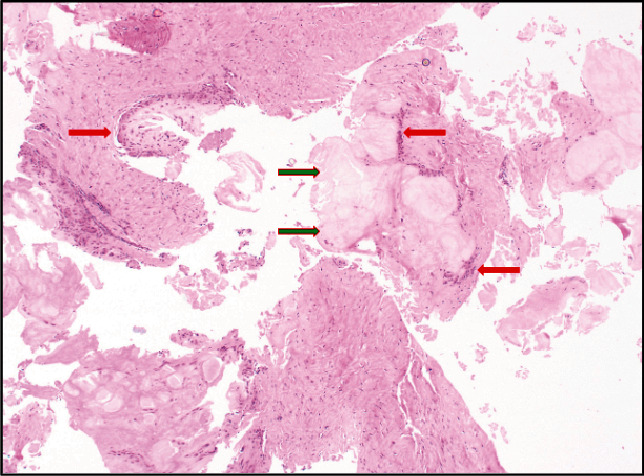
Light microscopy of central acellular amorphous pale eosinophilic material (green arrow) surrounded by histiocytes and multinucleated giant cells (red arrow). The central acellular amorphous pale eosinophilic material represents the area previously occupied by the monosodium urate crystals, which become dissolved in the histological staining process, and the surrounding histiocytes and multinucleated giant cells make up the foreign body granulomatous reaction that surrounds the previous location of the monosodium urate crystals, which are characteristic of gout. H&E stain. Magnification ×40.

The pathology report came back with findings that are demonstrated in Figure 3 and are consistent with gouty tophi. At the patient's initial postoperative visit 1 week after the procedure, he was informed of the intraoperative findings that were suspicious for gouty tophi, and he expressed surprise upon hearing this. The patient reported no history of any clinical gout flares. Furthermore, he had never been previously worked up for gout by his primary care provider or any other physician and had never taken any urate-lowering medications.

The patient continued to follow up with his orthopedic surgeon after his operative intervention. He initially wore a cam boot after his procedure. Next, he underwent a crutch wean protocol and partook in physical therapy. At his 7-month postoperative visit, he reported regaining a significant amount of his functional capacity with occasional soreness and minimal pain that was controlled with intermittent use of meloxicam.

At his 12-month postoperative visit, he reported no complaints and that he had completely regained all functionality in his left Achilles tendon. He stated that he was able to walk for as long as he pleased on his left ankle and that he was able to play golf again. He had a physical exam that was significant for a full range of motion in dorsiflexion, plantarflexion, abduction, adduction, inversion, and eversion at his left ankle. He had no overlying skin changes in the area of his prior gouty tophi or palpable deformities and had a less than 3 s capillary refill on his left first toe. Overall, the patient did quite well throughout his postoperative period. Of note, at his 12-month postoperative follow-up clinic visit, the patient reported no gout flare-ups or any other symptoms consistent with gout since undergoing the procedure.

## 3. Discussion

### 3.1. This Patient's Case in Relation to Similar Prior Reported Cases

This patient's case offers a unique and rare presentation of gouty tophi forming around retained, nonabsorbable suture material years after an Achilles tendon repair that appears to have only been documented three times previously in a review of the literature. Like this case, none of the prior patients in the other case reports reported a history of an acute gout flare. All of these prior cases differ from this patient in that they presented with an acute complaint of pain, erythema, and swelling surrounding the previous surgical site many years after their procedures for which they were prescribed antibiotics and had negative bacterial cultures. These patients were found to have an acute gout flare in the region where their tophi had formed that led to the failure of their initial surgical repair. Our patient underwent a revision procedure without any symptoms consistent with a gout flare and had tophi incidentally noted during his procedure. It is unknown if the tophi contributed to our patient's failure of his prior Achilles tendon repair or if they were incidentally found to be present and attached to the nonabsorbable sutures from his previous procedure. Even if the tophi did not directly contribute to the patient's need for a revision procedure, their presence indicates that the sutures from his prior surgical repair were likely to have progressively decreased integrity. The patient was likely also at a higher risk of developing an acute gout flare in the region of his previously repaired Achilles tendon in the same manner that the patients in the three previous case reports did [10–12].

Similar to these other three cases, nonabsorbable sutures were used in our patient's original Achilles tendon repair procedure. In our case and two of the prior cases, FiberWire nonabsorbable sutures were used, but in the other case, the suture's description was only noted to be nonabsorbable and did not have a particular suture brand listed [10–12]. Nonabsorbable sutures are essentially a foreign body that is permanently implanted inside of a patient's tendon repair site, and since they serve as a permanent implant, they can put patients at risk of developing the known sequelae of implants, such as the formation of biofilms by bacteria and the predisposition to infections [10]. It is evident from prior case reports that the formation of postsurgical gouty tophi should be considered in patients who are presenting with signs and symptoms concerning for an infectious process or gout flare, such as acutely severe pain, erythema, and swelling. The absence of a previous history of a gout flare-up should not exclude this rare presentation from the differential diagnosis, and the failure to respond to antibiotics should also lead clinicians to suspect this diagnosis. Even though our patient did not have an acute gout flare like these other patients, the presence of gouty tophi surrounding his sutures indicates that he was at a high risk for such a complication to occur [10–12].

The presence of gouty tophi has been noted postsurgically in tendons other than the Achilles tendon in case reports. Specifically, the flexor digitorum profundus and the extensor digitorum communis as well as the extensor indicis proprius tendons were noted to be the sites of formation of gouty tophi years after a previous surgical repair of the same tendons in two of the case reports. One of these patients presented with swelling, tightness, and triggering of the previously repaired tendon, and the other patient presented with a painful, palpable mass at the location of the previous tendon repair. Both of these cases involved the use of nonabsorbable sutures, specifically one used the brand Supramid and the other used a brand that was only noted to be nonabsorbable [13, 14]. These cases further indicate that nonabsorbable sutures may serve as a nidus for the attachment of gouty tophi as was seen in our case, but with so few cases previously documented in the literature, this cannot be stated with absolute certainty.

### 3.2. The Pathophysiology of Gouty Tophi and Foreign Body Granulomas

Foreign bodies in general can lead to the development of surrounding granulomas. From embedded glass to fish bones, there are many cases of retained foreign bodies that have led to the formation of granulomas [15, 16]. In a similar manner, retained suture material can serve as a foreign body. Furthermore, a gouty tophus is essentially the formation of a foreign body granulomatous reaction around monosodium urate crystals [17]. The pathophysiology of both the formation of gouty tophi in particular and foreign body granulomas in general is very similar. Essentially, the foreign bodies, whether they are monosodium urate crystals, nonabsorbable sutures, or another object, like a fish bone, serve as a nidus for an acute phase reaction that consists of neutrophils and inflammatory cytokines, such as TNF-*α*. The inability to clear the foreign body elicits a chronic, granulomatous response around the region of inflammation that has areas of fibrosis and vascularization as well as more of a lymphocyte-predominant immune response with T cells and B cells present. The primary difference between foreign body granulomas in general and gouty tophi is the presence of monosodium urate crystals in gouty tophi and their absence in other foreign body granulomas [15, 17]. A biopsy can differentiate between the two pathologies as tophi will demonstrate the presence of amorphous, eosinophilic material, representative of the monosodium urate crystals characteristic of gouty tophi, but a granuloma surrounding any other type of foreign body will not demonstrate those findings on histology [18].

While it is unknown exactly why gouty tophi may form in the location of a prior surgical intervention, a hypothesis can be made based on the underlying pathophysiology of tophi formation. A retained suture serves as a foreign body, and a foreign body serves as a nidus for the inflammatory reaction that leads to granuloma formation. That same pathophysiological process occurs whether the foreign body is a monosodium urate crystal or a different foreign body. Therefore, both monosodium urate crystals and other foreign bodies, like nonabsorbable sutures, can serve as a nidus for the development of an inflammatory granulomatous reaction. We hypothesize that while these processes can occur independently from one another and both lead to granuloma formation, it is actually the combination of monosodium urate crystals and retained, nonabsorbable sutures that leads to a more robust, clinically apparent inflammatory reaction greater than what monosodium urate crystals or retained sutures alone would be able to generate. What is likely occurring in these cases is that the patients have monosodium urate crystals that deposit throughout the body insidiously due to the underlying risk factors that lead to hyperuricemia, but their deposition on the retained suture material leads to a more robust granulomatous formation that manifests in clinically apparent tophi formation. While the significant gouty tophi formation did not lead to an acute gout attack in our case like it did in the three other Achilles tendon repair case reports that were complicated by gouty tophi formation, our patient's significant tophi formation may have contributed to the degenerative process that led to the breakdown of his prior surgical repair. Overall, in all four of these cases, including our case, the formation of clinically apparent gouty tophi surrounding the nonabsorbable, retained sutures was associated with a failure of the initial surgical repair and necessitated a revision procedure.

### 3.3. Hyperuricemia and Its Relationship to the Formation of Gouty Tophi

As mentioned in the introduction, the presence of hyperuricemia increases the likelihood of patients having a gout flare, but its absence does not exclude gout from the differential diagnosis. Of the three prior case reports of patients presenting with gouty tophi surrounding their nonabsorbable sutures years after an Achilles tendon repair procedure, one of the patients had an elevated uric acid level at the time of presentation, another had a normal uric acid level, and the third patient did not have their uric acid level measured [10–12]. Elevated levels of uric acid have been seen in case reports of postsurgical tophi surrounding nonabsorbable sutures for tendons other than the Achilles tendon [13, 14]. It is unknown if our patient had an elevated or normal uric acid level as outpatient review of his medical records from his primary care provider demonstrated that he had never had his uric acid level checked. Regardless, whether this patient had a normal or elevated uric acid level, his pathology findings were consistent with gouty tophi, and patients do not need to have an elevated uric acid level to be diagnosed with gout [8].

### 3.4. Potential Complications Associated With Postoperative Gouty Tophi Formation

The formation of gouty tophi around nonabsorbable suture material may result in the need for revision procedures and increase the chance of having an acute flare of gout around a previous tendon repair. Furthermore, the likelihood of these tophi serving as a nidus for an infection and its associated complications, such as the formation of an abscess, is increased. Gouty tophi can lead to the rupture of tendons, even in patients without a history of a tendon repair, so halting the formation of these tophi should be taken into consideration to prevent a potentially disastrous complication from occurring [19, 20]. Absorbable sutures have decreased tensile strength relative to nonabsorbable sutures, but they may also have decreased rates of rerupture and postoperative infections relative to nonabsorbable sutures [21]. Since nonabsorbable sutures may predispose an Achilles tendon repair site to the formation of gouty tophi, this potential complication should be taken into consideration when deciding which type of surgical suture is used. However, this cannot be stated definitively until future randomized controlled trials are performed to evaluate if nonabsorbable sutures truly increase the likelihood of gouty tophi formation. Our patient developed gouty tophi surrounding his previous Achilles tendon repair site in which nonabsorbable sutures were used, but he was able to escape the painful and acute sequelae that other patients with a similar presentation could not. Given that all three previously known cases of this rare presentation were found when the patients presented for an acutely painful episode, this patient was likely due to also have a similar episode as those patients in the future [10–12].

### 3.5. Potential Limitation and Bias

One potential limitation to this case report is the uncertainty regarding the patient's uric acid level. Since the patient followed up with his primary care physician at an outside institution in another region and despite best efforts by the surgical treating team, the evaluation of the patient's uric acid level is still unknown. While this has not hindered patient management, it would be beneficial to know in order to address the patient's potential underlying hyperuricemia that could precipitate a future clinical gout attack. A possible source of bias in this case report is that other differential diagnoses, such as pseudogout or calcium pyrophosphate deposition disease, were not seriously considered by the surgical team when the chalky substance was first noted in the operating room, but since biopsy results were confirmatory for gouty tophi, this possible source of bias did not seriously impede the outcomes of this case report.

## 4. Conclusion

The formation of gouty tophi around a prior Achilles tendon surgical site is very rare, and it has only been noted in the presence of retained, nonabsorbable sutures. While it is unknown if our patient's gouty tophi surrounding his retained suture material directly contributed to his need for a revision procedure, it is important to be aware of the potential complications that can result from these tophi, particularly the formation of an abscess or other infectious process in the region of the nonabsorbable sutures. While these cases are rare, appropriate clinical recommendations can be drawn from this case to guide providers who come across similar uncommon case presentations in the future. This case is a significant addition to the medical literature as it represents a rare postoperative side effect to Achilles tendon surgical repair with only three similar prior cases reported. Furthermore, this case is significant as many providers have never seen this clinical presentation in practice, and if it is untreated, it has the potential to lead to disastrous complications for patients, such as surgical site failure and significant infections. With the publication of this case report, the recognition of gouty tophi as a side effect of a prior Achilles tendon surgical site repair is more engrained in the literature and offers further evidence that the formation of these gouty tophi may be due to retained sutures that serve as a foreign body nidus for the attachment of the monosodium urate crystals that lead to pathological process known as gouty tophi formation.

For patients found to have gouty tophi surrounding their prior Achilles tendon surgical site repair, the clinical course of treatment is dependent on the patient's presentation. In our patient's case, there was chronic weakening of the patient's previously repaired Achilles tendon and loss of his ability to perform his activities of daily living, so a revision procedure was indicated to restore his mobility. We would recommend a similar operative course of treatment for patients presenting with chronic complaints associated with the formation of gouty tophi surrounding their prior Achilles tendon repair. Specifically, we would recommend a revision procedure for patients with chronic complaints, such as this one, who are no longer able to execute their activities of daily living and for patients with severe, lifestyle-altering pain. In contrast to this, if a patient presents with an acute complaint secondary to gouty tophi surrounding the retained sutures of their prior surgical repair like other case reports have documented, we recommend first treating the patient for an acute infectious process with antibiotics and surgical debridement as the failure to recognize and treat an infection could lead to the loss of the limb and potentially multiple-organ failure and death from sepsis. If a patient is treated for an infectious process but does not improve, the formation of gouty tophi must be considered on the differential diagnosis. We would recommend a biopsy of the affected region to definitively diagnose gouty tophi by histological evidence. Furthermore, we would recommend that all patients who are found to have the formation of gouty tophi around the sutures of their prior surgical site follow up with a primary care physician to have their uric acid levels checked and to potentially start urate-lowering medications, such as allopurinol, if clinically indicated. We also recommend these patients make appropriate lifestyle changes to lower the possibility of future acute gout flares, such as limiting alcoholic beverage consumption and avoiding foods that cause supersaturated levels of uric acid in the body, like seafood.

This case report calls attention to a widely unknown issue in a specific subset of patients undergoing orthopedic intervention and can inform future surgical and medical decision-making by highlighting this rare and uncommonly seen complication following Achilles tendon repair that has the potential to lead to disastrous complications. It highlights the potential negative sequalae that can result from the presence of gouty tophi around a prior surgical repair site, such as the increased risk of infectious complications and, furthermore, the potential failure of the prior surgical repair. Clinicians should be aware of this rare presentation that is potentially, but not certainly, linked to the use of nonabsorbable sutures, and they should consider it even in patients without a history of an acute gout flare. Future randomized controlled trials are needed to evaluate if nonabsorbable sutures are more likely to predispose patients to the formation of gouty tophi than absorbable sutures.

## Data Availability

The authors confirm that the data supporting the findings of this study are available within the article.
